# Water-pipe smoke condensate increases the internalization of Mycobacterium Bovis of type II alveolar epithelial cells (A549)

**DOI:** 10.1186/s12890-017-0413-7

**Published:** 2017-04-21

**Authors:** Esmaeil Mortaz, Shamila D. Alipoor, Masoud Movassaghi, Mohammad Varahram, Jahangir Ghorbani, Gert Folkerts, Johan Garssen, Ian M. Adcock

**Affiliations:** 1 Clinical Tuberculosis and Epidemiology Research Center, National Research Institute of Tuberculosis and Lung Diseases (NRITLD), Shahid Beheshti University of Medical Sciences, Tehran, Iran; 2 Department of Immunology, Faculty of Medicine, Shahid Beheshti University of Medical Sciences, Tehran, Iran; 3 Institute of Medical Biotechnology, Molecular Medicine Department, National Institute of Genetic Engineering and Biotechnology (NIGEB), Tehran, Iran; 4 Department of Biotechnology, School of Advanced Technologies in Medicine, Shahid Beheshti University of Medical Sciences, Tehran, Iran; 5 Department of Pathology and Laboratory Medicine, University of California, Los Angeles (UCLA), Los Angeles, CA USA; 6 Mycobacteriology Research Center (MRC) National Research Institute of Tuberculosis and lung Diseases (NRITLD), Shahid Beheshti University of Medical Sciences, Tehran, Iran; 7 Division of Pharmacology, Utrecht Institute for Pharmaceutical Sciences, Faculty of Science, Utrecht University, Utrecht, The Netherlands; 8 Nutricia Research Centre for Specialized Nutrition, Utrecht, The Netherlands; 9 Cell and Molecular Biology Group, Airways Disease Section, National Heart and Lung Institute, Imperial College London, Dovehouse Street, London, UK; 10 Priority Research Centre for Healthy Lungs, Hunter Medical Research Institute, The University of Newcastle, Newcastle, New South Wales Australia

**Keywords:** Water pipe, Endocytosis Activity, Type II Alveolar Epithelial Cells (A549)

## Abstract

**Background:**

Tuberculosis (TB) is a major global health problem, and there is an association between tobacco smoke and TB. Water pipe smoking has become an increasing problem not only in Middle Eastern countries but also globally because users consider it as safer than cigarettes. The presence of high levels of toxic substances in water-pipe smoke may be a predisposing factor that enhances the incidence of pulmonary disorders. For example, uncontrolled macropinocytosis in alveolar epithelial cells following exposure to water-pipe smoke may predispose subjects to pulmonary infection. Here, we studied the effects of water-pipe condense (WPC) on the internalization of Mycobacterium Bovis BCG by macropinocytosis in the alveolar epithelial cell line A549.

**Methods:**

A549 cells were exposed to WPC (4 mg/ml) for 24, 48, 72 and 96 h. Cell viability was studied using the methyl thiazolyldipenyl-tetrazolium bromide (MTT) reduction assay and proliferation by bromodeoxyUridine (BrdU) incorporation. Cells were exposed to FITC-Dextran (1 mg/ml) (as a control) and FITC-BCG (MOI = 10) for 20 min at 37 °*C before cells were* collected and the uptake of BCG-FITC determined by flow cytometry. Similar experiments were performed at 4 °*C as a control*. The Rho-associated protein kinase (ROCK) inhibitor Y-27632 (1 μM) was used to assess the mechanism by which WPC enhanced BCG uptake.

**Results:**

WPC (4 mg/ml) increased the uptake of BCG-FITC after 72 (1.3 ± 0.1 fold, *p* < 0.05) and 96 (1.4 ± 0.05 fold, *p* < 0.05) hours. No effect on BCG-FITC uptake was observed at 24 or 48 h. WPC also significantly increased the uptake of FITC-Dextran (2.9 ± 0.3 fold, *p* < 0.05) after 24 h. WPC significantly decreased cell viability after 24 (84 ± 2%, *p* < 0.05), 48 (78±, 3%, *p* < 0.05), 72 (64 ± 2%, *p* < 0.05) and 96 h (45 ± 2%, *p* < 0.05). Y-27632 completely attenuated the increased uptake of BCG by WPC. Cell proliferation showed a decreasing trend in a time-dependent manner with WPC exposure.

**Conclusion:**

WPC exposure increased epithelial cell endocytosis activity and death as well as enhancing their capacity for macropinocytosis. Our in vitro data indicates possible harmful effects of WPC on the ability of lung epithelial cells to phagocytose mycobacterium.

## Background

Mycobacterium tuberculosis (Mtb) is a critical threat to world health. Based on the 2015 WHO fact sheet, 10.4 million people were infected with TB, and 1.8 million died from the disease [1]. Mtb pathogenesis includes post-exposure effects on lung epithelial cells, including those lining the alveolar space [2]. The aerosolized bacterium can invade and bind to epithelial cells upon arrival into the alveolar space [3, 4], whereupon the alveolar epithelium cells internalize the Mycobacterium via macropinocytosis [3]. Macropinocytosis is an actin-mediated process, which enables non-selective uptake of soluble molecules. Macropinocytosis is associated with the formation of lamellipodia and membrane ruffling and is triggered by Mtb infection in alveolar type II epithelial cells [5].

Alveolar epithelial cells are crucial in providing the initial defense barrier against inhaled microorganisms in the respiratory system [2, 6] by producing antimicrobial peptides, reactive oxygen species (ROS), mucins, and other bacterial killing factors [7]. Moreover, these cells can act as antigen presenting cells to active resident CD8+ T-cells within the alveolar space. Therefore, alveolar epithelial cells play a critical role in the host defense against Mtb infection [8].

Water pipe (WP) smoking has a prevalence of 6 to 34% in Middle Eastern countries and has achieved a globally impact especially among adolescents, as it is considered safer than tobacco smoking [9, 10]. Previous studies on the chemistry of WP smoke have shown the presence of alarming levels of toxins such as nicotine, polycyclic aromatic hydrocarbons, and heavy metals. These may act as predisposing factors for several pathological states and may enhance the incidence of pulmonary disorders [11–15]. Even limited consumption of WP smoke is accompanied by a broad range of effects on human lung health [16]. For example, WP condensate (WPC) increased the expression of the matrix metalloproteinases (MMPs)-2 and −9 and that of the Toll-Like Receptor 4 (TLR4) in alveolar epithelial cells [17]. Up-regulation of these genes consequently affected the regulation of the immune response and pro-inflammatory reactions within the alveolar space [17].

The effect of cigarette and tobacco smoking on TB infection is well described [18–22]. In contrast, information regarding the effect of water-pipe smoke on TB is largely unknown. We hypothesized that WPC would have detrimental effects on the functions of lung alveolar epithelial cells. In this current study, we investigate the effect of WPC on the endocytosis activity of type II alveolar epithelial cell line (A549) against BCG in vitro.

## Methods

### Water-pipe smoke condensate (WPC) preparation

WPC was provided by Dr. El-Sabban (Beirut, Lebanon). Filters collected from water pipes after smoking sessions were stored in airtight containers at −20 °C. Smoke extract was prepared as described by Rammah et al. [17]. Filters were then washed with cell culture media (without fetal bovine serum) to elute off particulate mass. This material was stored at a concentration of 40 mg/ml. The resulting solution from any given smoking session was pooled and sterilized using 0.22-μm filters (Millipore, Munich, Germany).

### Cell culture and cell Viability assay

Human Type II alveolar epithelial cells (A549) (ATCC, USA) were grown in Roswell Park Memorial Institute medium (RPMI 1640) (Gibco; Carlsbad, CA, USA) supplemented with 10% heat-inactivated fetal bovine serum (FBS) (Gibco), 25 mM HEPES (Gibco), 100units/ml penicillin (Sigma, Munich, Germany), and 100ug/ml streptomycin (Sigma) in 5% CO_2_ at 37 °C.

Cells were seeded at a density of 10^4^ cells/cm^2^, and experimental procedures commenced when cells reached 70% confluence. Cells were exposed to 4 mg/ml WPC for 24, 48, 72 and 96 h. This dose was selected based on results from an earlier study [23]. The media was changed daily, and PBS was used in the control group. Cell viability was assessed 24, 48, 72 and 96 h after treatment with WPC using the colorimetric MTT metabolic activity assay [24]. A549 cells were seeded in a 96-well plate (Costar, Munich, Germany), and incubated in a CO_2_ incubator under the same WPC conditions as described above. At that time, the medium was refreshed, and 20 μl MTT solution (5 mg/ml in PBS) was added. The cells were incubated for another 4 h when the formazan crystals that formed were dissolved in dimethyl sulfoxide (100 μl) (Sigma) and the absorbance intensity measured at 490 nm with a reference wavelength of 620 nm. The relative cell viability (%) was expressed as a percentage relative to that seen in PBS-treated control cells at all-time points studied. All experiments were performed in triplicate.

### BrdU cell proliferation assay

A BrdU cell proliferation assay was performed to determine the effect of WPC on cell proliferation. Briefly, A549 cells were seeded at a density of 10^4^ cells/cm^2^ and incubated in a CO_2_ incubator. Cells were exposed to 4 mg/ml WPC or PBS control for 24, 48, 72 and 96 h. In each culture of different exposure times, cells were labeled with 5-bromo-2′-deoxyuridine (BrdU) for 4 h and the proliferation assay was performed according to the manufacturer’s instructions (Roche, Mannheim, Germany). The labeling medium was completely removed, 200 μl/well of Fixing/Denaturing solution was added to the cells, and they were incubated for a 15 min further at room temperature. Fixing/Denaturing solution was removed, 100 μl/well prepared conjugated anti-BrdU solution was added, and was incubated for 90 min at room temperature. The antibody conjugate was then removed, and the wells were rinsed three times with wash buffer. Finally, 100 μl/well substrate solution was added and incubated at room temperature for 30 min. The amount of BrdU incorporated into the newly synthesized DNA was measured at 450 nm with a reference wavelength of 690 nm using a microplate reader (Hercules, CA, USA). The data are presented as the measured absorbance based on the BrdU incorporation. The relative cell proliferation was expressed as a percentage relative to that seen in PBS-treated control cells at all-time points studied. Three independent experiments were performed with each sample being analyzed in triplicate.

### Uptake assay by fluorescein isothiocyanate (FITC)-Dextran

Confluent monolayers of A549 cells were either exposed to WPC or PBS as a control before being incubated with FITC-Dextran (Sigma-Aldrich) (1 mg/ml) for 20 min at 37 °C & 4 °C (as an additional control). After washing 2× with cold PBS, the cells were analyzed to evaluate the uptake of FITC-Dextran by flow cytometry (FACS Calibur, BD, USA) as described previously [25]. LPS (1000 ng/ml) was used as a positive control. Briefly, cells were washed and re-suspended in sodium acetate buffer (0.05 M; pH 4.5) containing 0.06% Trypan blue (Sigma) and incubated on ice for 5 min. A control group without Trypan blue was also analyzed in parallel. Staining with propidium iodide (PI) (Sigma-Aldrich) was performed to determine cell viability. The cells were re-suspended in 300ul PBS and prepared in TC tubes for reading on a flow cytometer.

Approximately, 10000 events were collected with a rate of 100–200 events/s and gated to eliminate the debris. Green fluorescence (480–530 nm) and red fluorescence (580–630 nm) were measured simultaneously in FL1 and FL3 channels, respectively. The fluorescence data were obtained at fixed gain setting in logarithmic (FL1) and in linear (FL3) mode. Data were analyzed using Flowing Software version 2.4.1 (Perttu Terho, Turku Centre for Biotechnology, Finland; www.flowingsoftware.com). The net uptake of FITC-Dextran was calculated by subtracting the total FL1 fluorescence intensity at 37 °C from the total FL-1 fluorescence intensity at 4 °C.

### Preparing FITC-BCG and cell infection

M. Bovis (BCG) was obtained as a gift from Pasteur Institute of Iran (IPI). To evaluate the internalization of FITC-BCG by flow cytometry, bacteria (BCG) were FITC-labeled by incubation with 0.5 mg/ml of FITC (Sigma-Aldrich) in 0.1 M carbonate buffer (pH = 9.0) for 2 h at 37 °C. FITC-BCG was then washed 3× with PBS to remove unbound FITC and cells were suspended in RPMI.

Confluent monolayers of A549 cells with or without WPC treatment were infected with labeled BCG or left uninfected as controls. Before infection, the labeled bacteria were opsonized by incubation with human AB^+^ serum (Pooled from 30 healthy male donors) as a source of complement components for 2 h at 37 °C. Cultures were infected with opsonized FITC-BCG at an MOI (Multiplicity of infection) =10 for 2 h at 37 °C or at 4 °C in a 5% CO_2_ environment followed by washing with 1× PBS to eliminate free organisms. Cytospins were performed and cells observed with a 40× objective lens by a fluorescent phase microscope equipped with a 520 nm filter for the FITC (Axiovert; ZEISS, Germany) or harvested to evaluate the level of infection by flow cytometry as early described.

### Incubation of the cultures in combination with Y-27632 and WPC

Confluent cell cultures were detached using trypsin (Sigma) and reseeded in 6-well plates. After 24 h, cultures were treated with WPC for an additional 72 h with a daily medium change. In some experiments, the Rho-associated protein kinase (ROCK) inhibitor Y-27632 (1 μM, Sigma-Aldrich) was added. After 72 h, an infection assay with FITC-labelled BCG was performed, and the rate of BCG uptake was analyzed by flow cytometry.

### Statistical analysis

All data were tested for normality by SPSS version 22 and analyzed for significance using analysis of variance and t- test (*P* < 0.05). The graphs were plotted using GraphPad Prism 6.

## Results

### WPC suppresses proliferation of A549 cells

**Fig. 1:**
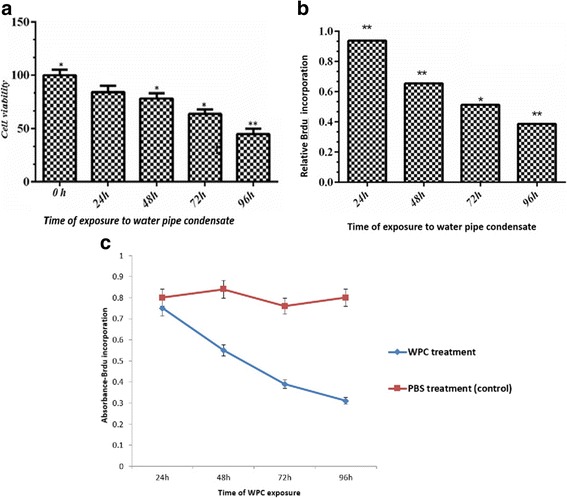
Water pipe condensate (WPC) suppress the viability and proliferation of A549 cells. Time-dependent effect of exposure of A549 cells to 4 mg/ml WPC on (**a**) cell viability as measured by MTT assay and cell proliferation as measured by BrdU assay (**b** & **c**). Data were analyzed at 24, 48, 72 and 96 h. The relative cell viability/proliferation (%) was expressed as a percentage relative to that seen in PBS-treated control cells at all time points studied (**c**). The data are presented as the measured absorbance based on the BrdU incorporation. Data represent mean ± S.E.M. of three independent experiments each repeated in triplicate. *P < 0.05, **P < 0.01 significantly different compared to control and ^^ *P* < 0.01 significantly different compared to control

The MTT metabolic viability assay was used to investigate the effect of WPC exposure on A549 cells after 24, 48, 72 and 96 h. A time-dependent suppression of cell viability by WPC (4 mg/ml) was observed with the greatest effect at 96 h with only 45% of cells viability (Fig. 1a).

BrdU analysis of cell proliferation was determined at all-time points. There was a trend towards decreased proliferation over time with WPC exposure (Fig. 1b and c). WPC decreased the relative proliferation rate in comparison to PBS-treated cells after 24, 48, 72 and 96 h exposure as follows: 0.93 ± 0.05%; 0.65 ± 0.03%; 0.51 ± 0.02% and 0.38 ± 0.01%, respectively (Fig. 1b). Proliferation was unaffected by PBS treatment, which had an average proliferation rate of 0.80 ± 0.03% over the time course of the experiment (Fig. 1c).

### WPC enhances uptake of FITC- Dextran by A549 cells

**Fig. 2:**
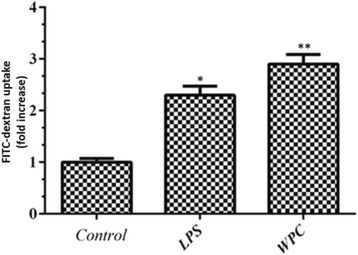
Water pipe condensate (WPC) increases the uptake of FITC-Dextran by A549 cells. WPC (4 mg/ml) enhances the uptake of FITC-Dextran by A549 cells after 24 h continuous exposure as determined by FITC-Dextran uptake assay. The effect of LPS (0.1 mg/ml) on uptake was measured as a positive control. Data are presented as mean ± S.E.M. of three independent experiments. *p < 0.05; **p < 0.01 versus control

To investigate the effect of WPC on uptake and macropinocytosis activity of A549 cells, an FITC-Dextran uptake assay was performed only at 24-h post treatment with WPC (4 mg/ml) since WPC did not affect cell viability at this time point. A flow cytometric fluorescence quenching assay was performed to quantify the internalized FITC-Dextran, and Trypan blue was used to quench the green fluorescence of bound particles to distinguish between cell-bound and internalized particles. In comparison to unexposed control cells, WPC was able to significantly increase the uptake of FITC-Dextran 2.9-fold (Fig. 2). This effect was greater than that seen with the positive control LPS which increased uptake in the treated cells 2.3-fold (Fig. 2).

### WPC enhances internalization of FITC-BCG by A549 cells

**Fig. 3:**
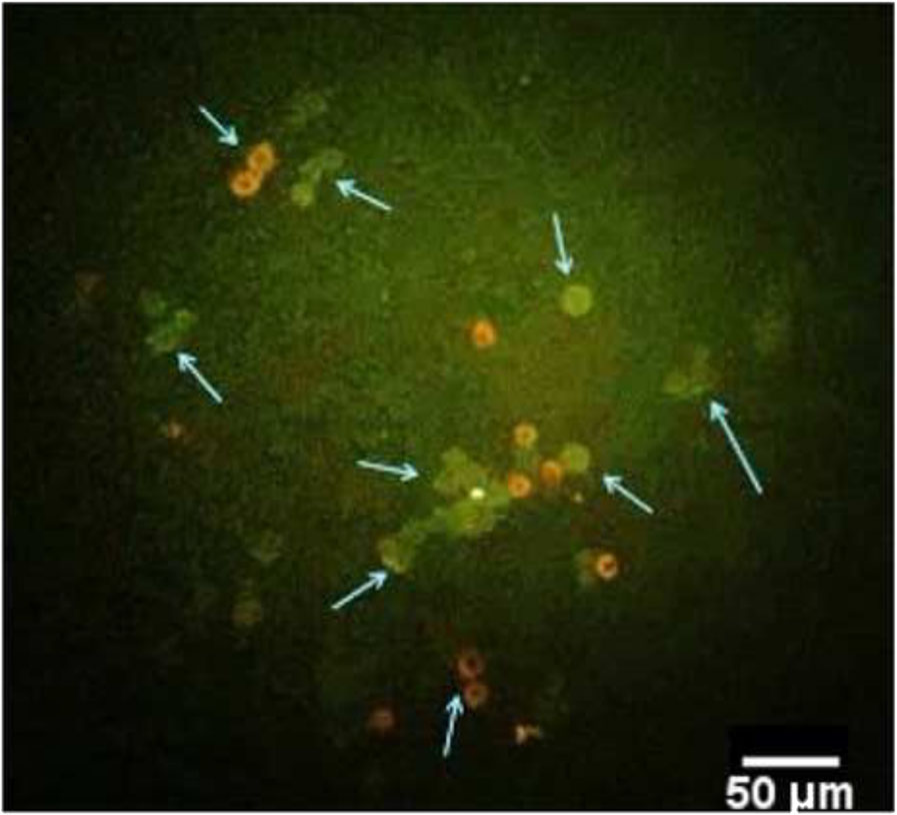
FITC-BCG binding and internalization by A549 cells. A549 cells were exposed to FITC-BCG for 20 min in standard culture conditions. After washing, the *green* surface fluorescence of bound bacteria was quenched for 3 min incubation on ice with Trypan *blue* and cytospun cells observed by fluorescence microscopy. The arrows point to the quenched cell-bound bacteria (*red* hollow circle) and internalized particles (*green*), which remain *green*, as they were not exposed to Trypan *blue*. The image was captured with a 40× objective and representing photo selected from 3 independent experiments

**Fig. 4:**
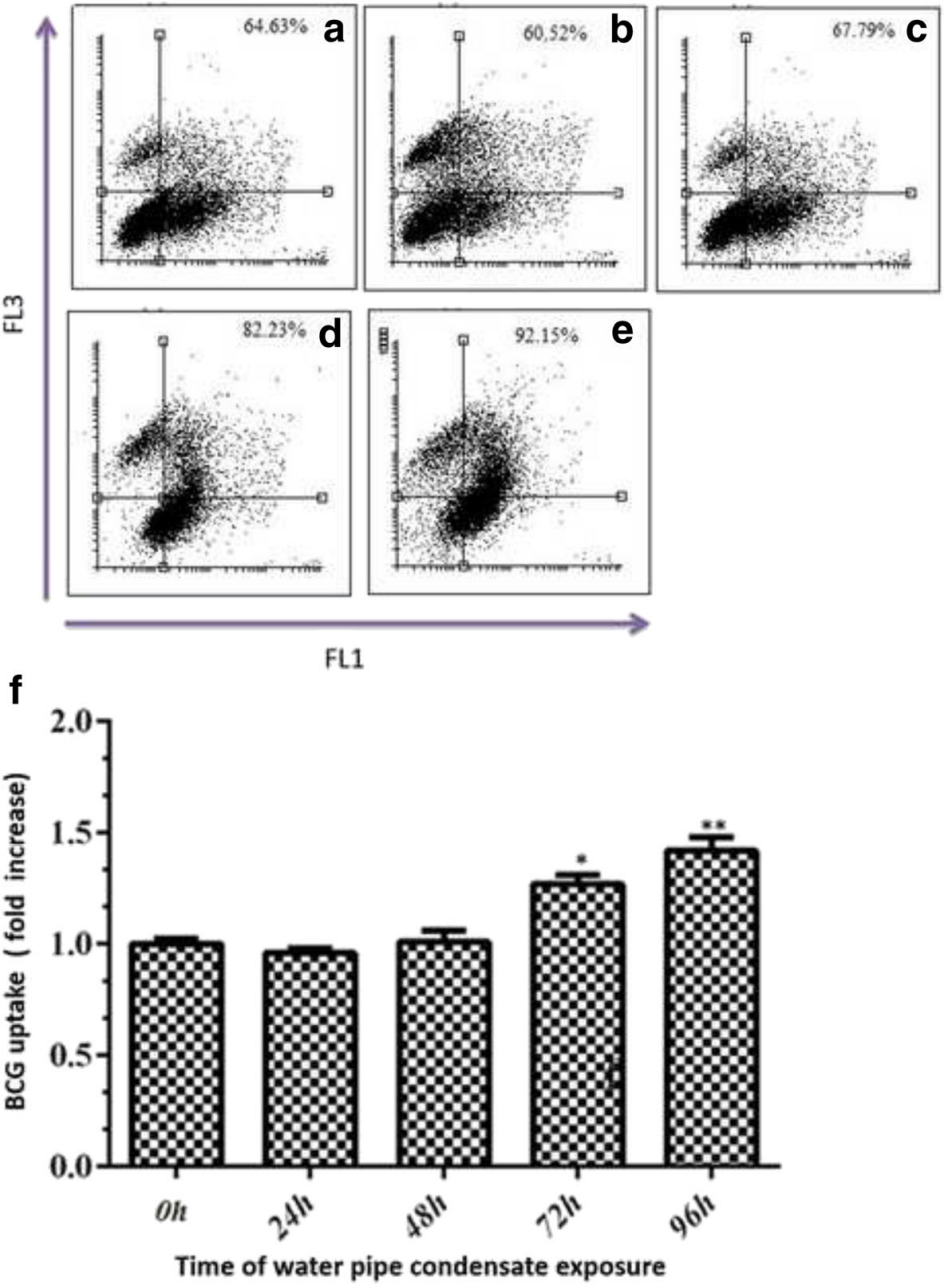
Time course of water pipe condensate (WPC) on the uptake of FITC-BCG. FITC-BCG uptake by A549 cells was increased in a time-dependent manner compared to PBS-treated cells. Uptake was increased 1.3- and 1.4-fold after 72 and 96 h exposure to WPC, respectively while no effect on uptake was seen after 24 and 48 h on cells. **a** PBS control; **b** 24 h; **c** 48 h; **d** 72 h; and **e** 96 h exposure. Data are presented of three independent experiments. The data are presented graphically in (**f**) which shows the percentages of FITC-BCG positive cells at different time points in response to WPC compared to PBS exposure. PBS exposure had no effect on uptake and time course data are presented relative to PBS control. All dot and bars plots results are presented as mean ± SD of the three independent experiments each repeated in triplicate. *p < 0.05; **p < 0.01 versus control was calculated

Internalization of FITC-labeled BCG was studied in exposed A549 cells. The results were investigated by fluorescent microscopy and also flow cytometry. By fluorescent microscopy, the bound bacteria were characterized as a red hollow circle because the green surface fluorescence of bound bacteria was quenched with trypan blue. On the other hand, the internalized bacteria, which were not exposed to trypan blue, appeared with green fluorescence (Fig. 3). Based on the result of the flow cytometric analysis; under control conditions, the uptake was 64.6 ± 2.51%. WPC incubation did not affect FITC-BCG uptake at 24 h and 48 h (60 ± 2.53 and 67.8 ± 2% respectively, Fig. 4b and c) In contrast, the uptake after 72 (D) and 96 (E) hours exposure was significantly increased to 82.2 ± 1% and 92.1 ± 2.6%, respectively. This indicates that WPC exposure increased the A549 cell infection rate by BCG 1.3- (1.3 ± 0.1, *p* < 0.05) and 1.4- (1.4 ± 0.05 *p* < 0.05) fold after 72 and 96 h, respectively (Fig. 4f), despite WPC causing a marked reduction in cell proliferation at the same time points (see Fig. 1).

### The Rho-Associated Kinase Inhibitor (Y-27632) attenuates WPC-Induced enhancement of BCG uptake by A549 cells

**Fig. 5:**
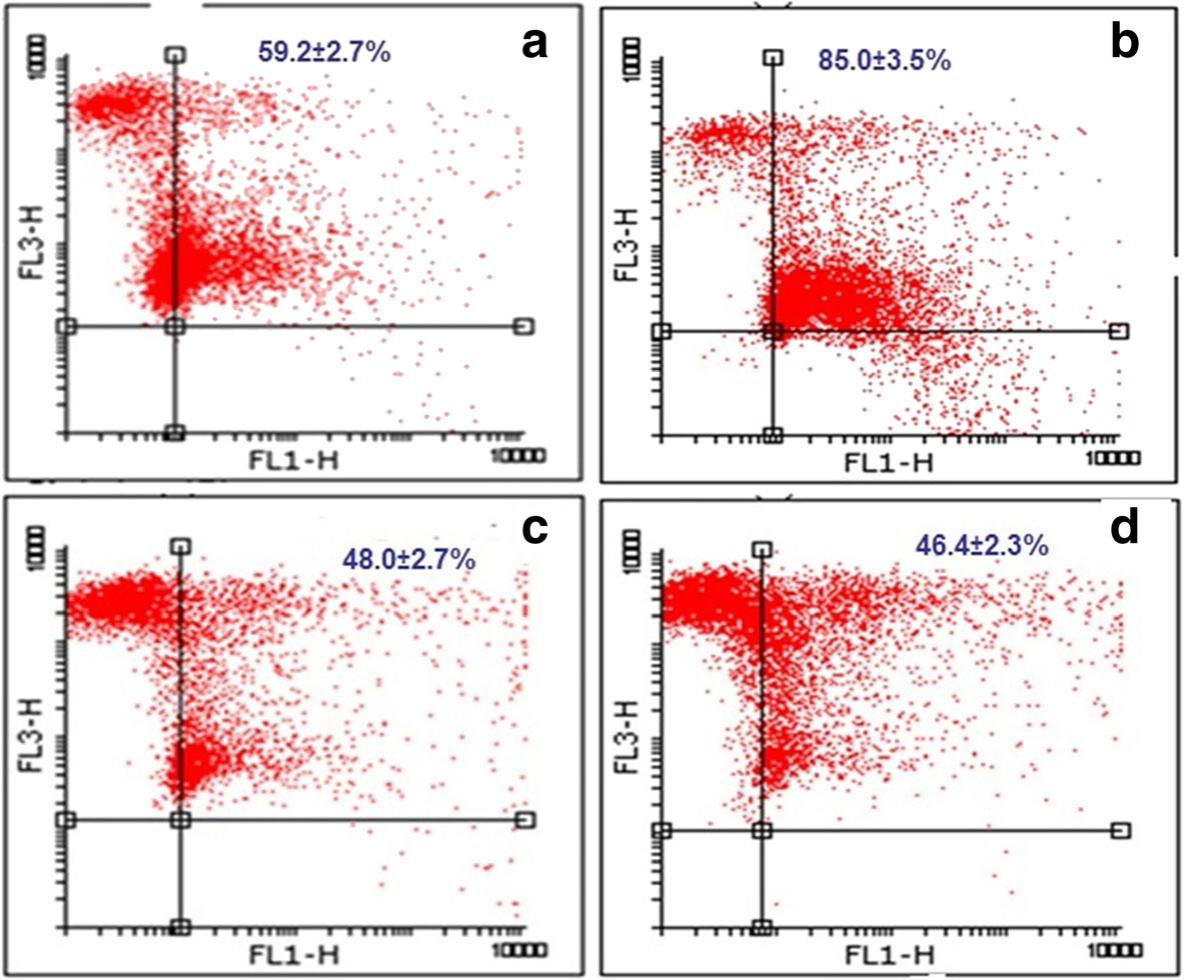
Involvement of the Rho-associated protein kinase (ROCK) pathway in water pipe condensate (WPC)-induced BCG uptake. The ROCK inhibitor Y-27632 (1 μM) attenuated the ability of WPC to enhance BCG uptake by A549 cells. Uptake of BCG by control PBS-treated cells (59.2 ± 2.7%) (**a**) was enhanced by water pipe condensate (WPC) exposure for 72 h (85.0 ± 3.5%) (**b**). Y-27632 pre-treatment shows a shift of cells back into FL1 in the presence of WPC (48.0 ± 2.7%) (**c**) while Y-27632 alone reduced to control levels of BCG uptake (46.4 ± 2.3) (**d**). The results are representative of 2 independent experiments

To examine whether the mechanism of WPC-induced BCG macropinocytosis involved the Rho/Rac pathway, we pre-treated the cells with the ROCK inhibitor Y-27632. Pre-treatment of WPC-exposed cells with Y-27632 attenuated the enhanced uptake of BCG seen with WPC alone, with labeled bacteria shifting back into FL1 compared to cells treated with WP alone (Fig. 5).

In comparison to BCG uptake in control, PBS-treated cells (59.2 ± 2.7%) (Fig. 5a), uptake was increased in cells exposed to WPC for 72 h (85.0 ± 3.5%) (Fig. 5b).

BCG uptake in WPC-exposed cells decreased to 48.0 ± 2.7% in the presence of Y-27632 (Fig. 5c). Y-27632 alone, in the absence of WPC, also attenuated basal BCG uptake (46.4 ± 2.3%) (Fig. 5d).

## Discussion

We studied the effects of WPC on alveolar epithelial cell function, specifically the effect of WPC on the endocytosis activity of A549 cells with BCG. We demonstrated that WPC exposure at a concentration of 4 mg/ml caused a time-dependent decrease in cell proliferation and viability from 24 h. The MTT assay reflects a reduction in metabolic activity available for proliferation, which may explain previous data indicating that WPC produced a concentration-dependent increase in the doubling time for A549 cell proliferation [23]. Our data also showed a significant increase in MTB uptake by A549 cells in the presence of WPC.

Alveolar epithelial cells are the first immunological barrier against Mtb following aerosol exposure [26]. Early studies reported that Mtb could invade and multiply in alveolar epithelial cells [4, 8], and as a consequence, these cells are thought to play a significant role in the initial immunological host response against Mtb [27, 28].

Rammah and colleagues have previously investigated the deleterious effects of WPC on endothelial cell function [23]. Moreover, WPC prevented endothelial cell proliferation by causing cell cycle arrest via the p53-p21 pathway without induction of apoptosis [29]. Despite many studies having been published regarding the effects of cigarette and tobacco smoke on alveolar epithelial cell function [20, 30–33], few studies have been performed using WPC. Further work on the mechanisms of the deleterious actions of WPC on the mechanisms underpinning the loss of cell function is required.

We also studied the phagocytic capacity of cells when treated with WPC. Thus, cells were incubated with WPC before exposure to BCG, and bacterial internalization was tracked by flow cytometry. As a control, we used FITC-Dextran to examine the effect of WPC on the macropinocytosis activity of A549 cells [34]. Many respiratory viruses or bacteria such as Mtb enter cells by this strategy [35]. Interestingly, WPC exposure increased the rate of dextran uptake in comparison to the effect of LPS [36–38], while WPC induced a higher rate of bacterial internalization. Thus, WPC may increase macropinocytosis by alveolar epithelial cells. This was further supported by the fact that exposure to WPC induced a higher rate of BCG infection in alveolar epithelial cells. Mtb infection has previously been reported to require macropinocytosis for internalization to epithelial cells [35]. In addition, WPC increases the secretion of matrix metalloproteinase in alveolar epithelial cells [15], emphasizing the multi-faceted detrimental effects of WPC.

Mycobacteria can survive and replicate in epithelial cells, in macrophages, and in the alveolar spaces of the lung [3, 8]. Infected alveolar epithelial cells undergo cellular necrosis, which results in the release of mycobacteria [39]. Thus, alveolar epithelial cells may aid mycobacterial dissemination during primary infection [39]. Apoptosis is suppressed in infected alveolar epithelial cells, and necrotic cell death occurs, which is a consequence of increased permeation of cell membrane and internalization of live bacilli [40].

Exposure of alveolar epithelial cells to WPC change their capacity for macropinocytosis, which may lead to increased cell permeability to infectious agents such as BCG. Infection of A549 may trigger host immune responses and the onset of inflammatory cytokine secretion, which recruits immune inflammatory cells to the site of infection. It cannot be excluded that WP consumption may contribute to a higher incidence of TBC infections.

Although the invasion of epithelial cells by MTB has been demonstrated [3, 28, 41, 42], the mechanisms of mycobacterial attachment or internalization in these nonprofessional phagocytes have not been elucidated [3, 41]. Garcıa-Perez and colleagues [41] demonstrated that MTB induced redistribution of actin filaments, the formation of lamellipodia, and increase fluid phase uptake in A549 cells. These data together suggest that MTB internalization is due to macropinocytosis [41]. Further evidence for micropinocytosis is derived from the role of the small RhoG GTPase, which promotes membrane ruffling and macropinocytosis [43]. Previous studies have already shown the effectiveness of smoking on the Rho GTPase [44].

Water-pipe smoking may enhance macropinocytosis internalization activity in epithelial cells through other pathways [42]. Syndecan 4 on alveolar epithelial cells acts as a receptor for the MTB mycobacterial heparin-binding hemagglutinin adhesin [42]. Activation of syndecan 4 stimulates signaling pathways such as RhoG and Rac1, leading to micropinocytosis [45]. Previous studies have reported effects of smoking on Rac1 activation [46, 47] or GTPase mediated pathways [43]. Our results showed that WPC enhanced the uptake of BCG by A549 cells and that this uptake was suppressed by the ROCK inhibitor Y-27632 via prevention of bacterial internalization by macropinocytosis.

There are some limitations to this study. Ideally, we would have liked to confirm these data obtained in A549 cells in primary human alveolar type II cells. However, such cells are difficult to obtain, as they require fresh isolation from human lung tissue. Rodent type II cells are more readily available but show functional differences from human type II cells. Furthermore, although we have demonstrated that the enhanced uptake of MTB in these experiments is probably due to micropinocytosis, it is unclear whether this is the primary effect of WPC on these cells. In addition, our data on cell survival and proliferation cannot rule out the effect of WPC on A549 cell death. As such, the primary effect of WPC on the epithelial uptake on MTB remains to be elucidated.

## Conclusion

In conclusion, WPC treatment can enhance alveolar epithelial cell uptake of BCG by macropinocytosis in a time-dependent manner. This occurs at the same time as epithelial cells undergo metabolic inhibition and reduced survival and proliferation highlighting the adverse effects of WPC on alveolar epithelial cell function.

## Abbreviations

BCG: Bacillus Calmette–Guérin (BCG) Vaccine
COPD: Chronic Obstructive Pulmonary Diseases
FITC: Fluorescein Isothiocyanate
MMP: Matrix Metalloproteinases
Mtb: Mycobacterium Tuberculosis
MTT: Thiazolyldipenyl-Tetrazolium Bromide
PI: Propidium Iodide
ROS: Reactive Oxygen Specious
TB: Tuberculosis
WPC: Water Pipe Condense
