# Correction to: Water-pipe smoke condensate increases the internalization of Mycobacterium Bovis of type II alveolar epithelial cells (A549)

**DOI:** 10.1186/s12890-020-01244-1

**Published:** 2020-09-22

**Authors:** Esmaeil Mortaz, Shamila D. Alipoor, Masoud Movassaghi, Mohammad Varahram, Jahangir Ghorbani, Gert Folkerts, Johan Garssen, Ian M. Adcock

**Affiliations:** 1 Clinical Tuberculosis and Epidemiology Research Center, National Research Institute of Tuberculosis and Lung Diseases (NRITLD), Shahid Beheshti University of Medical Sciences, Tehran, Iran; 2 Department of Immunology, Faculty of Medicine, Shahid Beheshti University of Medical Sciences, Tehran, Iran; 3 Molecular Medicine Department, Institute of Medical Biotechnology, National Institute of Genetic Engineering and Biotechnology (NIGEB), Tehran, Iran; 4 Department of Biotechnology, School of Advanced Technologies in Medicine, Shahid Beheshti University of Medical Sciences, Tehran, Iran; 5 Department of Pathology and Laboratory Medicine, University of California, Los Angeles (UCLA), Los Angeles, CA USA; 6 Mycobacteriology Research Center (MRC) National Research Institute of Tuberculosis and lung Diseases (NRITLD), Shahid Beheshti University of Medical Sciences, Tehran, Iran; 7 Division of Pharmacology, Faculty of Science, Utrecht Institute for Pharmaceutical Sciences, Utrecht University, Utrecht, The Netherlands; 8 Nutricia Research Centre for Specialized Nutrition, Utrecht, The Netherlands; 9 Cell and Molecular Biology Group, Airways Disease Section, National Heart and Lung Institute, Imperial College London, Dovehouse Street, London, UK; 10 Priority Research Centre for Healthy Lungs, Hunter Medical Research Institute, The University of Newcastle, Newcastle, New South Wales Australia

**Correction to: BMC Pulm Med 17, 68 (2017)**

**https://doi.org/10.1186/s12890-017-0413-7**

Following publication of the original article [1], the authors that there is an error in the FACs plots detailed in Fig. 4.

The error is that the plot of panel ‘C’ has been duplicated as the plot of panel ‘A’.

Please see the corrected figure in this correction article.

The authors apologize for any inconvenience caused.

**Fig. 4:**
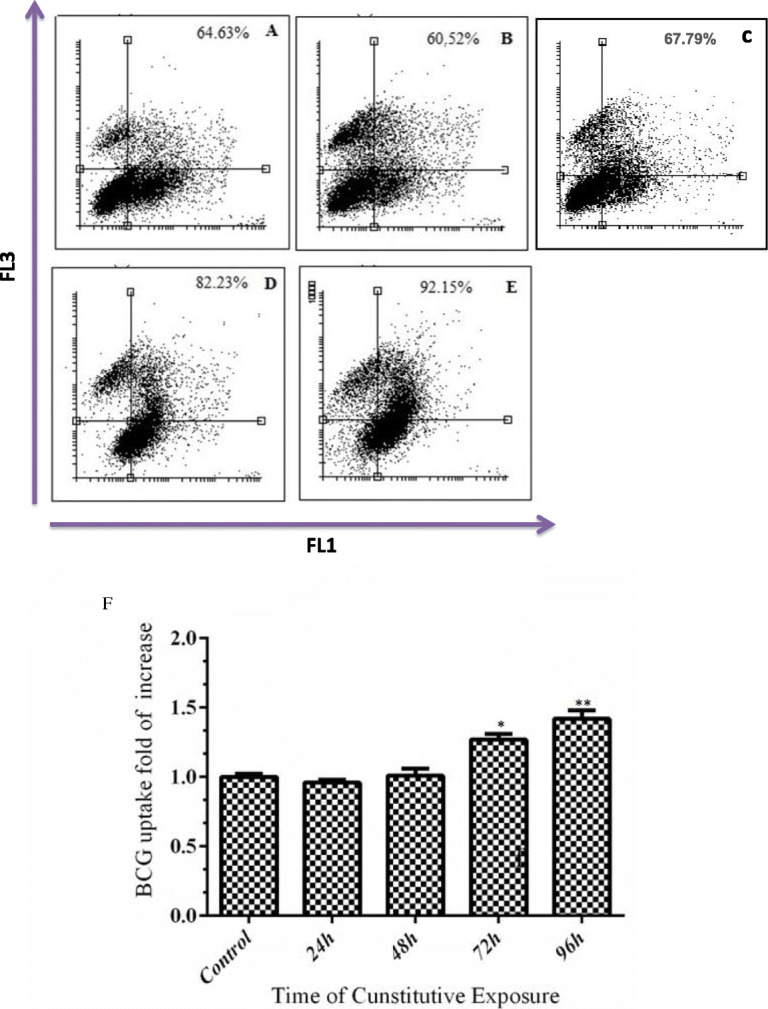
Time course of water pipe condensate (WPC) on the uptake of FITC-BCG. FITC-BCG uptake by A549 cells was increased in a time-dependent manner compared to PBS-treated cells. Uptake was increased 1.3- and 1.4-fold after 72 and 96 h exposure to WPC, respectively while no effect on uptake was seen after 24 and 48 h on cells. **a** PBS control; **b** 24 h; **c** 48 h; **d** 72 h; and **e** 96 h exposure. Data are presented of three independent experiments. The data are presented graphically in (**f**) which shows the percentages of FITC-BCG positive cells at different time points in response to WPC compared to PBS exposure. PBS exposure had no effect on uptake and time course data are presented relative to PBS control. All dot and bars plots results are presented as mean ± SD of the three independent experiments each repeated in triplicate. *p < 0.05; **p < 0.01 versus control was calculated
